# Depletion of Adipose Stroma-Like Cancer-Associated Fibroblasts Potentiates Pancreatic Cancer Immunotherapy

**DOI:** 10.1158/2767-9764.CRC-24-0298

**Published:** 2025-01-02

**Authors:** Joseph Rupert, Alexes Daquinag, Yongmei Yu, Yulin Dai, Zhongming Zhao, Mikhail G. Kolonin

**Affiliations:** 1 Center for Metabolic and Degenerative Diseases, The Brown Foundation Institute of Molecular Medicine for the Prevention of Disease, McGovern Medical School, Houston, Texas.; 2 Center for Precision Health, McWilliams School of Biomedical Informatics and School of Public Health, The University of Texas Health Sciences Center at Houston, Houston, Texas.

## Abstract

Immune checkpoint blockade therapy, transformative in some cancer types, has remained ineffective for patients with pancreatic cancer. The effects of subpopulations of cancer-associated fibroblasts (CAF) on cancer progression and therapy resistance are incompletely understood. In this study, the roles of CAFs expressing platelet-derived growth factor receptor β (*Pdgfrb*) and of CAFs expressing markers of adipose stromal cells (ASC) were analyzed in mice with pancreatic ductal adenocarcinoma. Ablation of *Pdgfrb*+ cells resulted in suppression of primary pancreatic tumor growth, reduction of extracellular matrix deposition, and increased cancer cell metastasis to the liver. A peptide D-CAN, which induces apoptosis in ASC-like CAFs, also reduced pancreatic tumor growth and extracellular matrix deposition while promoting metastases. Single-cell RNA sequencing demonstrated that depletion of either *Pdgfrb+* or ASC-like CAFs decreased frequencies of tumor endothelial cells and viable cancer cells. However, whereas depletion of *Pdgfrb+* CAFs led to stronger induction of cancer cell aggressiveness markers, depletion of ASC-like CAFs had an opposite effect on remaining CAFs. Depletion of ASC-like CAFs using D-CAN also led to higher infiltration of cytotoxic T-lymphocytes and B-lymphocytes. Administration of anti-PDL1 antibody (aPDL1), which inhibits the immune checkpoint, had a stronger suppressive effect on tumor growth when combined with D-CAN in both female and male mice. Liver metastases were also reduced by the D-CAN/aPDL1 combination more effectively than by aPDL1 alone in female mice. We conclude that improved approaches to target ASC-like CAFs may be effective in combination with immunotherapy.

**Significance:**

This study shows that populations of CAFs have distinct effects on pancreatic cancer progression and shows that depletion of CAFs expressing adipose markers potentiates tumor/metastasis suppression effects of immune checkpoint blockade.

## Introduction

Pancreatic cancer is the third deadliest cancer in the United States. The median survival time of patients with pancreatic ductal adenocarcinoma (PDAC) is 6 to 12 months (1). Patients mostly present with disseminated disease, and the majority of patients are offered palliative chemotherapy because immune checkpoint blockade therapy, transformative in some cancer types, has remained ineffective for PDAC (2). The tumor microenvironment (TME) plays an important role in the progression to aggressive disease (3). Cancer-associated fibroblasts (CAF) are a heterogeneous and plastic population of nonmalignant mesenchymal tumors cells that are recruited early in cancer and evolve as disease progresses to modulate tumor growth, metastatic dissemination, chemotherapy resistance, and immunosuppression (4). There are two major subtypes of CAFs observed in carcinomas, one exhibiting a matrix-producing contractile phenotype and the other an immunomodulatory phenotype, termed myCAFs and iCAFs, respectively (5). In PDAC, CAFs most proximal to the cancer cells exhibit high α smooth muscle actin (αSMA) expression that is associated with high TGFβ signaling, which promotes differentiation of contractile CAFs (myCAFs) and inhibits differentiation of inflammatory CAFs (iCAFs) via suppression of the IL1 receptor (6). In addition, CAFs that express MHC II and CD74, termed “antigen-presenting CAFs” (apCAF), have been described (5, 7).

It has been proposed that CAFs can arise from local organ-resident fibroblasts and transdifferentiate from other cell types (4). In animal models of PDAC, pancreatic stellate cells have turned out to contribute minimally to CAFs, and the origin of CAFs conferring PDAC prometastatic and chemoresistant properties seems to be distinct (8). Increased aggressiveness of pancreatic and other carcinomas is associated with obesity, the condition of white adipose tissue (WAT) overgrowth (9). WAT has been uncovered as a source of CAFs (10) that retain the gene expression signature of normal adipose stromal cells (ASC), the mesenchymal stromal cells of WAT (11). Our studies in mouse models have shown that ASCs recruited by tumors and becoming CAFs promote cancer progression (12). They have revealed a role of these ASC-like CAFs in epithelial–mesenchymal transition induction and chemotherapy resistance (13, 14). The role of ASC-like CAFs in immune evasion and their effect on immunotherapies has not been explored.

## Materials and Methods

### Murine model of PDAC

All mouse experiments were approved by and performed in accordance with the University of Texas Health Science Center at Houston Institutional Animal Care and Use Committee. Mice were housed in a barrier facility with *ad libitum* access to food and water and were maintained on a twelve-hour light/dark cycle. All mice used in experiments were made obese by feeding a high-fat diet (D12451i; Research Diets, Inc.) for 24-week prior to experiments. KPC FC1242 cells originally isolated from the LSL-KrasG12D:LSL-Trp53R172H:Pdx1-Cre genetic murine model of PDAC (15) were orthotopically implanted into mice as described (16). Briefly, 12-month-old obese mice were anesthetized using inhaled isoflurane, subcutaneously injected with Ethiqa XR (3 mg/kg), and placed in lateral recumbency on their right side. The mice were shaved and aseptically prepped. A small incision was made into the abdomen, and the pancreas and spleen retracted. Using a 28G needle and a 1-mL syringe, KPC cells were injected into the pancreas (100,000 cells/100 μL PBS) over a period of 30 second. The abdominal musculature was sutured, and the skin was closed using metal wound clips.

### Mouse treatment

Depletion of *Pdgfrb-*expressing cells was achieved using the Pdgfrb-TK mouse strain, which has thymidine kinase expression controlled by the *Pdgfrb* promoter, inducing apoptosis in *Pdgfrb+* cells upon administration of ganciclovir (GCV). GCV (Sigma-Aldrich, Cat. #G2536) was intraperitoneally injected at 50 mg/kg every other day for 25 days in Pdgfrb-TK mice as described (17). D-CAN, the ASC-homing cyclic peptide CSWKYWFGEC (18) linked via aminohexanoic acid with an apoptosis-inducing peptide KFAKFAKKFAKFAK, was synthesized from D-amino acids by AmbioPharm (14, 19). Lyophilized D-CAN was reconstituted using PBS and subcutaneously injected at 30 mg/kg every other day in C57BL/6J mice (#000664; Jackson Laboratories) for 25 days. Antibody against anti-PDL1 (aPDL1; clone 10F.9G2 #BE0101; BioXCell) was intraperitoneally injected twice a week at 10 mg/kg in PBS in C57BL/6J mice. Vehicle control mice received injections of PBS. IVIS imaging was performed as described using Xenogen (14). Mice were euthanized by cervical dislocation, and tumors were excised and weighed using an analytical scale. All mice were included into analyses.

### Tissue analysis

Portions of tumors were fixed in 10% neutral buffered formalin for 72 hours for paraffin embedding, and tumor cross-sections (5 μm) were cut for histology. Hematoxylin/eosin staining was performed as described (13). Trichrome staining was performed using a commercially available kit (HT15-1KT; Sigma-Aldrich). Tumors and livers were individually minced and digested with collagenase and dispase. Cell suspensions were strained and processed for single-cell RNA sequencing (scRNA-seq) analysis (tumor) or metastasis quantification (liver). An aliquot of 200,000 cells was taken from each tumor single-cell suspension and pooled according to treatment (i.e., PBS, GCV, and D-CAN) for a total of 600,000 cells per treatment group. For each mouse, one half of the right liver lobe was removed and fixed in 10% neutral buffered formalin, whereas the remaining liver in its entirety was digested into a single-cell suspension. To quantify metastases, individual liver cell suspensions were normalized by plating (1 × 10^6^ cells/well) in triplicate in 6-well plates in DMEM (SH30243.01; Cytiva) supplemented with 20% FBS for 7 days and fixed using cooled methanol (−20°C) for 20 minutes, and colonies were visualized using crystal violet stain. Samples were blinded for analysis.

### scRNA-seq

scRNA-seq was performed using 8,000 cells and 31,000 reads for each treatment group. As previously described (13), single-cell capture and library construction were performed with Chromium Single Cell 3ʹ Reagent Kit v3.1. Barcoded single-cell gel beads were loaded onto Chromium Next GEM ChipG (PN-1000120). After running on 10× Chromium Single-Cell Controller, gel beads in emulsion were generated. The barcoded and full-length cDNAs were produced after incubation of the gel beads in emulsion and amplified via PCR. Library was qualified by Agilent Bioanalyzer 2100 and quantified by RT-PCR on QuantStudio3. Sequencing was performed with the Illumina NextSeq 550 System using High Output Kit v2.5 (50,000 reads per cell). The Cell Ranger Single-Cell Software Suite v.3.1.0 was used for bioinformatic analysis. The reads were aligned to the mouse transcriptome reference (mm10, Ensembl 93) with STAR. Raw read count tables were analyzed using the Seurat (v3.1.1) pipeline on R platform (3.5.2). FindVariableGenes was used to calculate the principal components. Cell clusters were identified using the Shared Nearest Neighbor algorithm with a resolution parameter of 0.8. Uniform Manifold Approximation and Projection for Dimension Reduction and t-distributed stochastic neighbor embedding clusters of cells were identified based on the first 10 principal components, and feature plots were displayed with the log (raw read count +1) of gene/cell.

### Statistical analysis

Microsoft Excel and GraphPad Prism were used to graph data as the mean ± SD and to calculate *P* values using the homoscedastic Student *t* test for comparison of two groups or one-way ANOVA for comparison of three or more groups. *P* < 0.05 was considered significant. The total sample size was at least *N* = 3 per group, and experiments were repeated at least twice with similar results.

### Data availability

The datasets generated for this study can be found in the Gene Expression Omnibus database GSE256025, Token onmruiyuxjqrfyr. Other data generated in this study are available upon request from the corresponding author.

## Results

### Depletion of *Pdgfrb+* cells suppresses extracellular matrix deposition and tumor growth but promotes metastasis

**Figure 1:**
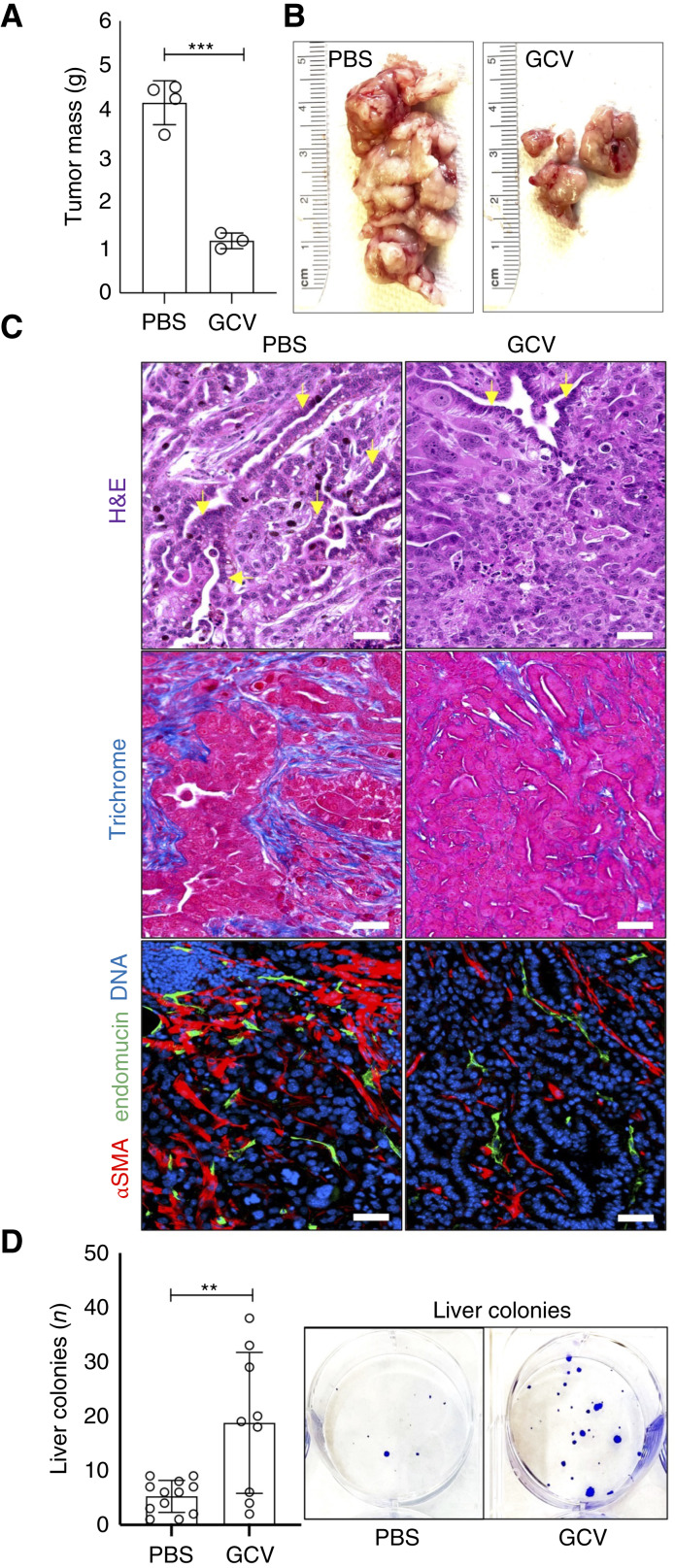
The effect of *Pdgfrb*+ CAF depletion on orthotopic PDAC progression. Tissues resected from *Pdgfrb-TK* male mice after 25 days of GCV (*N* = 3) or control PBS (*N* = 4) treatment were analyzed. **A,** KPC tumor size upon resection. **B,** Representative resected KPC tumors. **C,** Paraffin sections of tumors in **B** stained with hematoxylin/eosin, trichrome blue, or subjected to αSMA and endomucin (endothelial marker) immunofluorescence. Arrows indicate examples of well-differentiated KPC cells forming cancerous ducts. Scale bar, 50 μm. **D,** Quantification of KPC cells in livers based on colony counts. Images: representative wells. *, *P* < 0.05; **, *P* < 0.01; ***, *P* < 0.005; Student *t* test. H&E, hematoxylin and eosin.

To investigate the role of *Pdgfrb-*expressing stromal cells in the TME, we utilized the KPC orthotopic model of PDAC (16). To establish the effect of stromal cell depletion during tumor growth, *Pdgfrb-TK* males were orthotopically grafted with KPC cells and immediately received i.p. injections of PBS or GCV every other day for 25 days and then euthanized (Supplementary Fig. S1A and S1B). At euthanasia, *Pdgfrb-TK*/GCV mice had smaller tumor mass compared with *Pdgfrb-TK*/PBS mice (Fig. 1A and B). Increased necrosis and worse tumor differentiation, the ability of tumor epithelial cells to resemble pancreatic ductal cells and form cancerous ducts, has been linked with increased metastasis (20). Tumors from *Pdgfrb-TK*/GCV mice displayed more necrosis and less differentiated tumors than tumors from control mice (Fig. 1C; Supplementary Fig. S1C). As apparent from trichrome staining, *Pdgfrb-TK*/GCV mice had lower collagen deposition in the tumor stroma compared with control mice (Fig. 1C). Tumors from *Pdgfrb-TK*/GCV mice also had a reduction in stroma expressing αSMA (Fig. 1C). There were no visible metastases in the liver upon gross observation. However, liver single-cell suspensions plated in culture allowed quantification of metastatic cells [Fig. 1D (right)]. There were significantly more colonies formed from livers of *Pdgfrb-TK*/GCV mice versus controls (Fig. 1D).

To determine if cancer progression was affected by acute necrosis and inflammation caused by short-term depletion of *Pdgfrb+* cells, we used the same model to test the long-term effects of *Pdgfrb*+ cell depletion. *Pdgfrb-TK* mice received 13 cycles of GCV or PBS pretreatment over 25 days, left to recover for 2 months, and then orthotopically grafted with KPC cells (Supplementary Fig. S1D and S1E). For *Pdgfrb-TK*/GCV pretreated mice, tumor growth was also decreased compared with control mice 25 days after implantation (Supplementary Fig. S1F).

### Depletion of ASC-like CAFs suppresses extracellular matrix deposition and tumor growth

**Figure 2:**
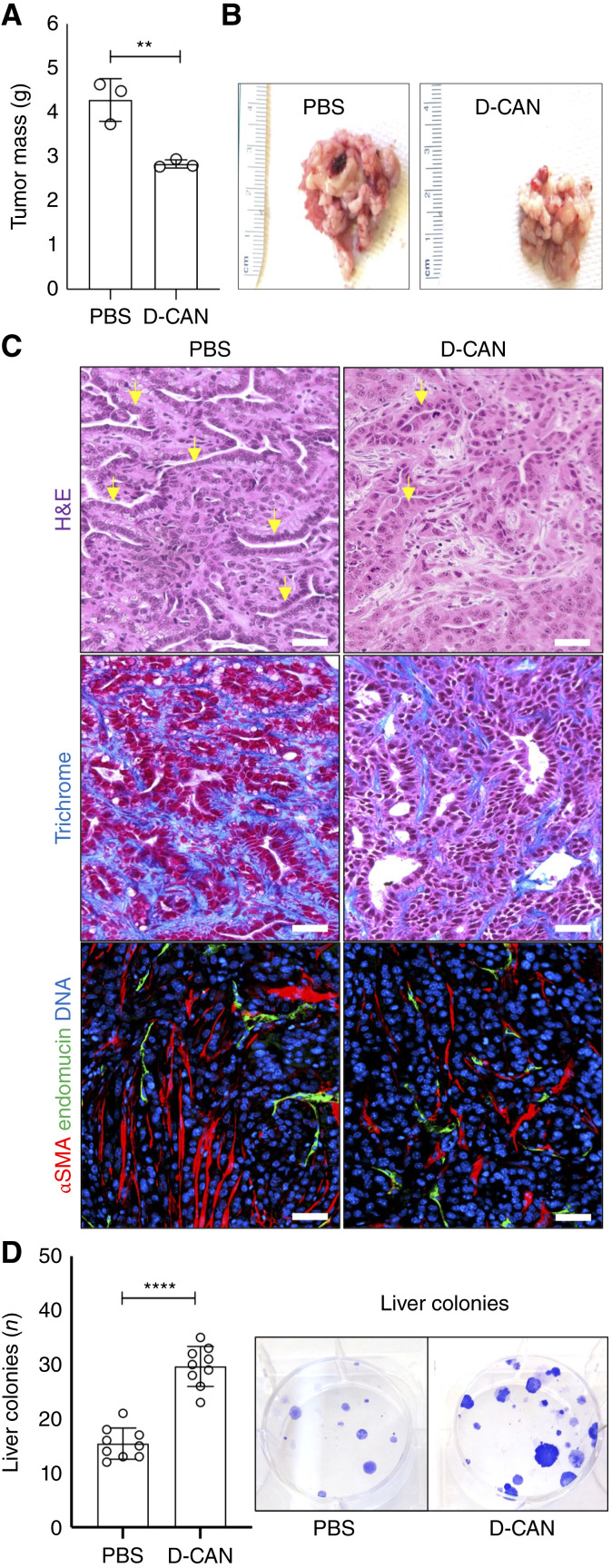
The effect of ASC-like CAF depletion on orthotopic PDAC progression. Tissues resected from male mice after 25 days of D-CAN (*N* = 3) or control PBS (*N* = 3) treatment were analyzed. **A,** KPC tumor size upon resection. **B,** Representative resected KPC tumors. **C,** Paraffin sections of tumors in **B** stained with hematoxylin/eosin, trichrome blue, or subjected to αSMA and endomucin immunofluorescence. Arrows indicate examples of well-differentiated KPC cells forming cancerous ducts. Scale bar, 50 μm. **D,** Quantification of KPC cells in livers based on colony counts. Images: representative wells. *, *P* < 0.05; **, *P* < 0.01; ****, *P* < 0.001, Student *t* test. H&E, hematoxylin and eosin.

We next investigated the effects of ablating CAFs expressing markers of ASCs previously shown to be recruited by carcinomas (21). We used a hunter–killer peptide D-CAN, which specifically homes to and induces apoptosis primarily in ASCs and their derivatives expressing nonglycanated decorin and PDGFRβ (14, 19). KPC cells were orthotopically implanted into male mice, which then received s.c. injections of PBS or D-CAN every other day for 25 days and were then euthanized (Supplementary Fig. S1G and S1H). At euthanasia, mice treated with D-CAN had smaller tumor mass than control mice (Fig. 2A and B). Mice treated with D-CAN also had decreased differentiation of tumor epithelial cells (Fig. 2C; Supplementary Fig. S1I). Depletion of ASCs resulted in reduction of collagen deposition in the tumor stroma, as evident from trichrome staining (Fig. 2C). Tumors from D-CAN–treated mice had a reduction in cells expressing αSMA (Fig. 2C). Liver metastases were not visible by gross observation. However, metastatic dissemination quantified via liver cell plating [Fig. 2D (right)] revealed significantly higher cancer cell numbers in livers of D-CAN–treated mice (Fig. 2D).

### Depletion of *Pdgfrb+* and ASC-like CAFs has distinct effects on PDAC and the TME

**Figure 3:**
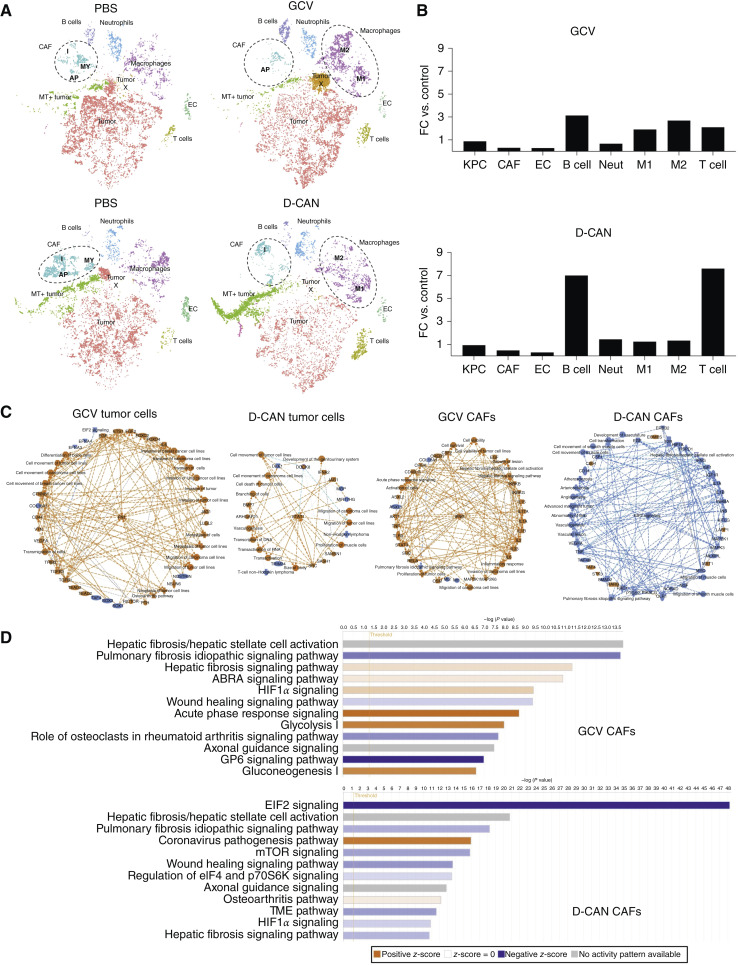
The effects of *Pdgfrb*+ CAF vs. ASC-like CAF depletion on the TME. Shown are representative analyses of tumors from Figs. 1 and 2. **A,** t-distributed stochastic neighbor embedding clusters with cell populations identified based on heatmap analysis (not shown) show changes in labeled populations. **B,** Changes in frequencies of cells corresponding to labeled populations in **A**. **C,** IPA analysis of scRNA-seq data of gene expression in KPC cells and CAFs from (**A**) identifying top genes and pathways induced (orange) or suppressed (blue). **D,** Quantification of top pathways’ induction (orange) or suppression (blue) in CAFs from **A**. AP, Antigen Presenting CAF; FC, fold change; MY, myCAFS; Neut, neutrophil.

Next, we compared the effects of depleting *Pdgfrb-*expressing CAFs and ASC-like CAFs on cell populations and gene expression in the PDAC TME using scRNA-seq to analyze tumors from *Pdgfrb-TK* mice and D-CAN–treated mice. Data were collectively analyzed using unsupervised clustering and t-distributed stochastic neighbor embedding. After annotation based on gene expression profiles unique to certain cell types was performed, nine clusters were assigned (Fig. 3A and B). In addition to the majority of KPC tumor cells (tumor), a subcluster of KPC cells with high expression of genes linked with cancer aggressiveness was detected (tumor X). Another KPC subcluster had high expression of monocyte markers and mitochondrial genes (MT + tumor), suggesting these cells to be dying cancer cells engulfed by macrophages. CAFs roughly corresponded to previously reported subclusters, iCAFs, myCAFs, and apCAFs. Other major clusters were identified as endothelial cells (EC), macrophages, B cells, T cells, and neutrophils. In both models of CAF depletion, there was a decrease in CAFs: −3.2-fold for *Pdgfrb-TK*/GCV and −2.1-fold for D-CAN. There was also a decrease for ECs (−3.4-fold for *Pdgfrb-TK*/GCV and −3.25-fold for D-CAN). In contrast, there were increases in B cells (+3.1-fold for *Pdgfrb-TK*/GCV and +7-fold for D-CAN) and T cells (+2.1-fold for *Pdgfrb-TK*/GCV and +7.6-fold for D-CAN; Fig. 3A and B). Immunofluorescence with CD3 and endomucin antibodies confirmed the D-CAN effects on T cells (Supplementary Fig. S2A) and the endothelium (Supplementary Fig. S2B), respectively. According to markers for inflammatory and alternative polarization, both M1 and M2 macrophages were increased in *Pdgfrb-TK*/GCV (M1 +1.9-fold; M2 +2.7-fold) and D-CAN–treated (M1 +1.3-fold; M2 +1.3-fold) mice (Fig. 3A and B).

We also compared the different effects of targeting *Pdgfrb+* and ASC-like CAFs on the transcriptome of specific subsets of CAFs and ECs in tumors by ingenuity pathway analysis (IPA) using scRNA-seq data. In *Pdgfrb-TK/GCV* mice, the remaining CAFs had reduced expression of iCAF and myCAF markers and increased expression of apCAF markers (Fig. 3A). In contrast, in mice treated with D-CAN, the remaining CAFs had reduced expression of myCAF and apCAF markers, whereas iCAF markers were increased (Fig. 3A). Differentially expressed genes (FDR < 0.05) from combined CAF and cancer cells were analyzed by IPA to map differentially regulated signaling networks (Fig. 3C). For *Pdgfrb-TK/GCV* mice, CAF analysis revealed an increase in activation of pathways involving metastasis and inflammation, including “migration of cell lines,” “proliferation of tumor cells,” “invasion of carcinoma cell lines,” and “inflammatory response” (Fig. 3C). Network analysis of KPC cells from *Pdgfrb-TK/GCV* mice also showed increased activity of signaling pathways involved in metastasis, including “invasion of tumor cell lines,” “invasion of breast cancer cell lines,” “metastasis of tumor cells,” “migration of tumor cells,” “movement of tumor cell lines,” “cell movement of carcinoma cell line,” “invasion of carcinoma cells,” and “transmigration of cells” (Fig. 3C). In contrast, CAFs from D-CAN–treated mice showed an overall suppression activity for pathways involved in “angiogenesis” and “vascularization” (Fig. 3C). Whereas KPC cells from D-CAN–treated mice still displayed activation of pathways “cell movement of carcinoma lines,” “cell movement of tumor cell lines,” and “migration of tumor cell lines,” it is noteworthy that activation of the “cell death of cancer cells” pathway was detected (Fig. 3C). The top 12 regulated pathways after IPA analysis for CAFs depict an overall activation of pathways involving fibrosis, acute phase response signaling, and glycolysis in *Pdgfrb-TK*/GCV mice and a decrease in pathways related to fibrosis and translation in D-CAN–treated mice (Fig. 3D). Interestingly, predicted activity of the hypoxia-inducible factor-1α pathway, known to have a role in metastasis, was different between *Pdgfrb-TK*/GCV mice (activated) and D-CAN–treated (suppressed) mice (Fig. 3D). Volcano plots generated using IPA (FDR < 0.05, log_2_ fold change > 0.59) illustrate various significantly regulated genes associated with metastasis (Supplementary Fig. S2C). The corresponding lists of the top 10 upregulated and down-egulated genes based on fold change are shown in Supplementary Fig. S2D.

### D-CAN potentiates anti-PDL1 antibody efficacy in PDAC

**Figure 4:**
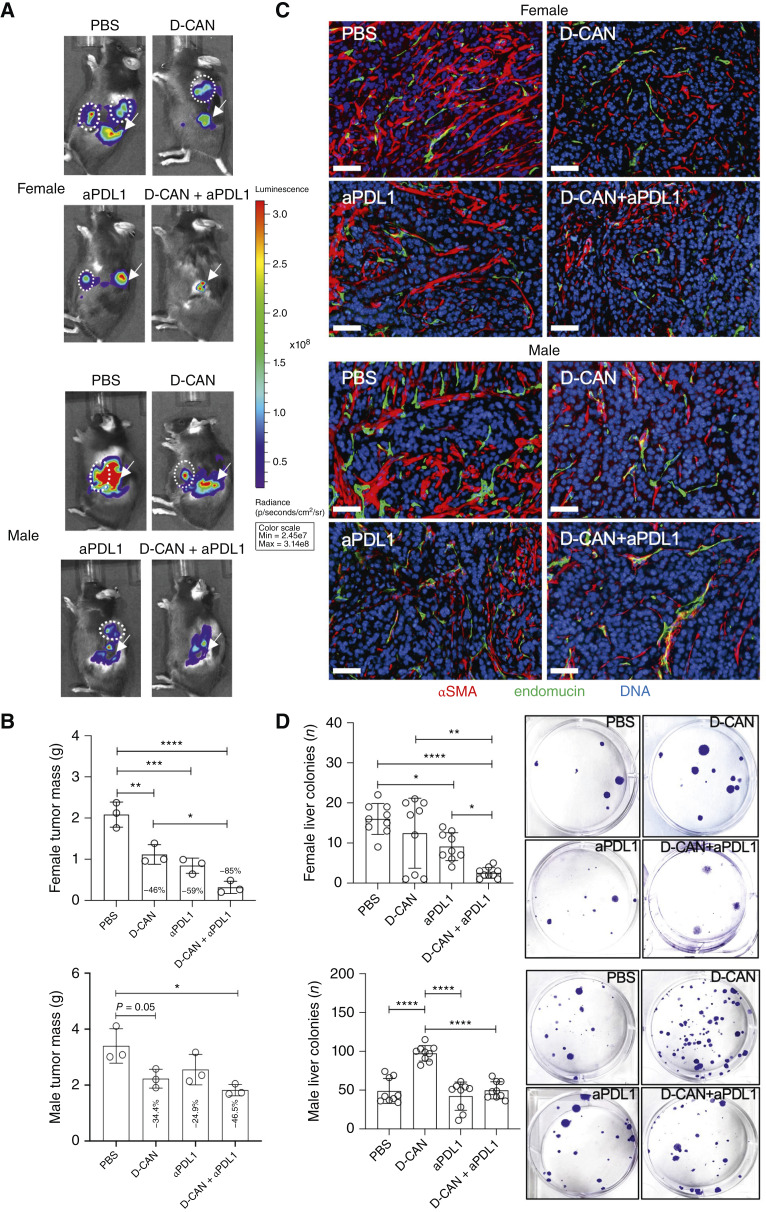
ASC-like CAF depletion synergizes with immune checkpoint blockade. Following orthotopic grafting of KPC-Luc cells, female (*N* = 3 per group) and male (*N* = 3 per group) mice received PBS, D-CAN, aPDL1, or the combination D-CAN + aPDL1. **A,** IVIS images of two representative mice at day 22 showing the location of pancreatic primary tumor graft (arrow) and extrapancreatic area of tumor invasion and metastases (punctate). **B,** KPC tumor size upon resection from females and males. **C,** Paraffin sections of representative tumors subjected to αSMA and endomucin immunofluorescence. Scale bar, 50 μm. **D,** Quantification of KPC cells in livers based on colony counts. Images: representative wells. *, *P* < 0.05; **, *P* < 0.01; ***, *P* < 0.001; ****, *P* < 0.0001, ANOVA. Max, maximum; Min, minimum.

Because we observed a substantial increase in infiltrating T cells in mice treated with D-CAN (Fig. 3A and B), we investigated the effects of combining D-CAN with an antibody blocking PDL1 (aPDL1). KPC cells genetically engineered to express luciferase were orthotopically implanted into obese female and male mice. Immediately following tumor cell implantation, PBS, D-CAN, aPDL1, or D-CAN + aPDL1 combination treatments were initiated. S.c. injections of D-CAN were given every other day, and aPDL1 antibody was administered intraperitoneally twice a week (Supplementary Fig. S3A and S2B). Mice were injected with luciferin at day 22 after tumor implantation to visualize tumor location and metastasis *in vivo* (Fig. 4A). Compared with other treatment groups, mice receiving D-CAN, aPDL1, or D-CAN + aPDL1 had reduced luciferase luminescence in the implantation area (Fig. 4A, arrows). At euthanasia, 25 days after tumor implantation, female treatment groups were confirmed to have statistically smaller tumors versus PBS controls (Fig. 4B). Notably, D-CAN + aPDL1 treatment resulted in the largest tumor size reduction (−85%) versus D-CAN (−34.4%) and aPDL1 (−24.9%). For males, tumor mass was reduced in D-CAN + aPDL1–treated mice versus PBS-treated mice (−46.5%), and although not statistically significant, tumor size was reduced in D-CAN and aPDL1 groups versus the PBS group (Fig. 4B). Compared with PBS controls, reduced numbers of aSMA+ cells were observed in D-CAN–treated and aPDL1-treated mice, with the largest reduction being observed in the D-CAN+ aPDL1 combination treatment for both females and males (Fig. 4C).

Finally, we quantified metastatic dissemination in these animals. Separate luminescence areas corresponding to metastases were observed for PBS control, D-CAN–treated, and aPDL1 groups but not for the D-CAN + aPDL1 combination group (Fig. 4A, dashed). Confirming an additive effect on metastasis, there were significantly fewer metastatic colonies recovered from liver cell suspensions for the D-CAN + aPDL1 combination group than for the PBS control group in both females and males (Fig. 4D). Interestingly, whereas D-CAN alone induced metastases in males, confirming data in Fig. 2D, this was not observed for females (Fig. 4D). The D-CAN + aPDL1 combination resulted in the lowest numbers of metastatic cells, significantly lower than for the D-CAN alone group for both females and males (Fig. 4D). Treatment with nonimmune IgG isotype control for aPDL1 did not have an effect with or without D-CAN (Supplementary Fig. S3C and S3D). Importantly, for female mice, the D-CAN + aPDL1 combination resulted in numbers of metastatic cells recovered being significantly lower compared with aPDL1 monotherapy (Fig. 4D).

## Discussion

It has remained debated whether inactivation of CAFs may present therapeutic benefits. Depletion of CAFs expressing αSMA (17) or of Sonic Hedgehog–dependent CAFs in mice promoted metastases and decreased survival (22). Moreover, inflammatory CAFs seem to enable cytotoxic T-cell infiltration and potentiate immunotherapy (23). However, CAFs accompanying cancer evolution to aggressive stages (11) may be a favorable drug target in the context of other anticancer treatments.

Studies by independent groups have identified a novel population of CAFs, which are ASC-like, in mouse and human carcinomas (11, 24). Our results demonstrate that depletion of *Pdgfrb-*expressing or ASC-like stromal cells suppresses extracellular matrix deposition and growth of primary tumors. They also indicate important and distinct roles for *Pdgfrb+* and ASC-like CAFs in the regulation of PDAC progression to metastasis. It should be noted that in the Pdgfrb-TK model, GCV also targets other proliferating *Pdgfrb+* cell types, such as a specific subset of pericytes, which may have their own specific role in tumor progression. Similarly, D-CAN also targets pericytes and other stromal cells that express PDGFRβ and nonglycanated decorin (14, 18, 19). Nonetheless, our findings are consistent with a previous report based on breast cancer models in which metastases were activated by CAF depletion (17). These effects are likely due to those CAFs supporting ECs and, hence, preventing hypoxia-driving cancer invasiveness. Targeting of either *Pdgfrb+* or ASC-like CAFs resulted in increased activity of signaling networks related to cancer aggressiveness in cancer cells, indicating their partial overlap. However, the two treatments had distinct effects on the subsets of CAFs. Changes in CAFs were largely influenced by IFNγ in *Pdgfrb-TK*/GCV mice and by eukaryotic initiation factor 2 in D-CAN–treated mice [Fig. 3C (right)]. Differences in cancer cell responses were also observed between *Pdgfrb-TK*/GCV mice and D-CAN–treated mice. Changes in cancer cells were largely influenced by fibronectin-1 in *Pdgfrb-TK*/GCV mice and signal transducer and activator of transcription 1 in D-CAN–treated mice [Fig. 3C (left)]. Finally, *Pdgfrb+* CAF and ASC-like CAF depletion had different effects on immune cells. Together, these data suggest that ASC-like CAFs play a unique role in the TME. Our results show that ablating ASC-like CAFs synergizes with aPDL1 in reducing tumor growth and increases the effectiveness of this immune checkpoint blockade therapy in suppressing PDAC metastases to the liver in female mice. This is consistent with previous findings showing that aPDL1 efficacy in the KPC PDAC model is linked with decreased presence of aSMA+ CAFs (25). Cancer progression to metastasis was not inhibited by the combination treatment to the same extent in males. The reason for this sex-specific difference remains to be determined. It is possible that this is due to male mice becoming more obese on high-fat diet and having comparatively more aggressive KPC tumor growth.

In conclusion, our study established the role of ASC-derived CAFs in PDAC progression to metastasis and defines their function in controlling the immune TME. Whereas CAF depletion suppressed tumor growth, metastases were induced, possibly because of increased tumor hypoxia. Therefore, the potential safety of CAF targeting as a monotherapy remains questionable. Our data generate a groundwork for future experiments investigating unique subpopulations of CAFs and suggest that approaches to CAF modulation may lead to improved efficacy of immunotherapy in PDAC and other refractory cancers.

## Supplementary Material

Figure S1 A, Fig. 1 schematic of testing the effect of Pdgfrb+ CAF depletion on PDAC progression. B, initial body weight in experiment A. C, experiment A tumor section H&E staining showing inflammation (i), necrosis (n), and well-differentiated tumor cells (arrows). D, schematic of testing the effect of Pdgfrb + CAF pre-depletion on PDAC progression. E, initial body weight in experiment D. F, tumor weights in experiment D. G, Fig. 2 schematic testing the effect of ASC-like CAF depletion on PDAC progression. H, initial body weight in experiment G. I, experiment G tumor section H&E staining indicating necrosis (n) and well-differentiated tumor cells (arrows). Scale Bars = 500μm. *=p<0.05.

Figure S2 The effects of Pdgfrb+ CAF vs ASC-like CAF depletion on the TME. A, tumor section immunofluorescence for CD3 (Red) expression to measure T-cell infiltration and nuclei (blue) (left), CD3+ cell quantification (right). B, tumor section immunofluorescence for endomucin (green) to quantify endothelial cell density and nuclei (blue) (left), endomucin quantification (right). In A-B, for each tumor, a minimum of 15 fields (10X) were taken and quantified using Image J. Images were converted to 8-bit and a threshold was applied to only highlight endomucin expression and percent area was measured. An average was calculated for all images per tumor. Scale bar=100μm. *=p<0.05, ***=p<0.001.C, Volcano plots generated by IPA analysis of scRNAseq data in Fig. 3 identifying top genes induced (red) or suppressed (green) in KPC cells and CAFs from mice subjected to Pdgfrb+ CAF or ASC-like CAF depletion. D, Lists of the most upregulated (top) and downregulated (bottom) genes.

Figure S3 A, Fig. 4 schematic testing if D-CAN synergizes with immune checkpoint blockade (aPDL1) in male and female mice. B, Initial body weights in experimental mice. C, Tumor weight from an experiment in female mice investigating the effects of D-CAN / aPDL1 with non-immune IgG and IgG + D-CAN data combined with Fig. 4B and 4D data. *=p<0.05, **=p<0.001, ***=p<0.0001. D, Metastatic liver tumor colony assay from mice in C.
