# Supplementary figures and images for: Depletion of Adipose Stroma-Like Cancer-Associated Fibroblasts Potentiates Pancreatic Cancer Immunotherapy
Source: Cancer Res Commun. 2025 Jan 2;5(1):5–12. doi: 10.1158/2767-9764.CRC-24-0298 (PMC11694247; doi:10.1158/2767-9764.CRC-24-0298)

## Slide 1
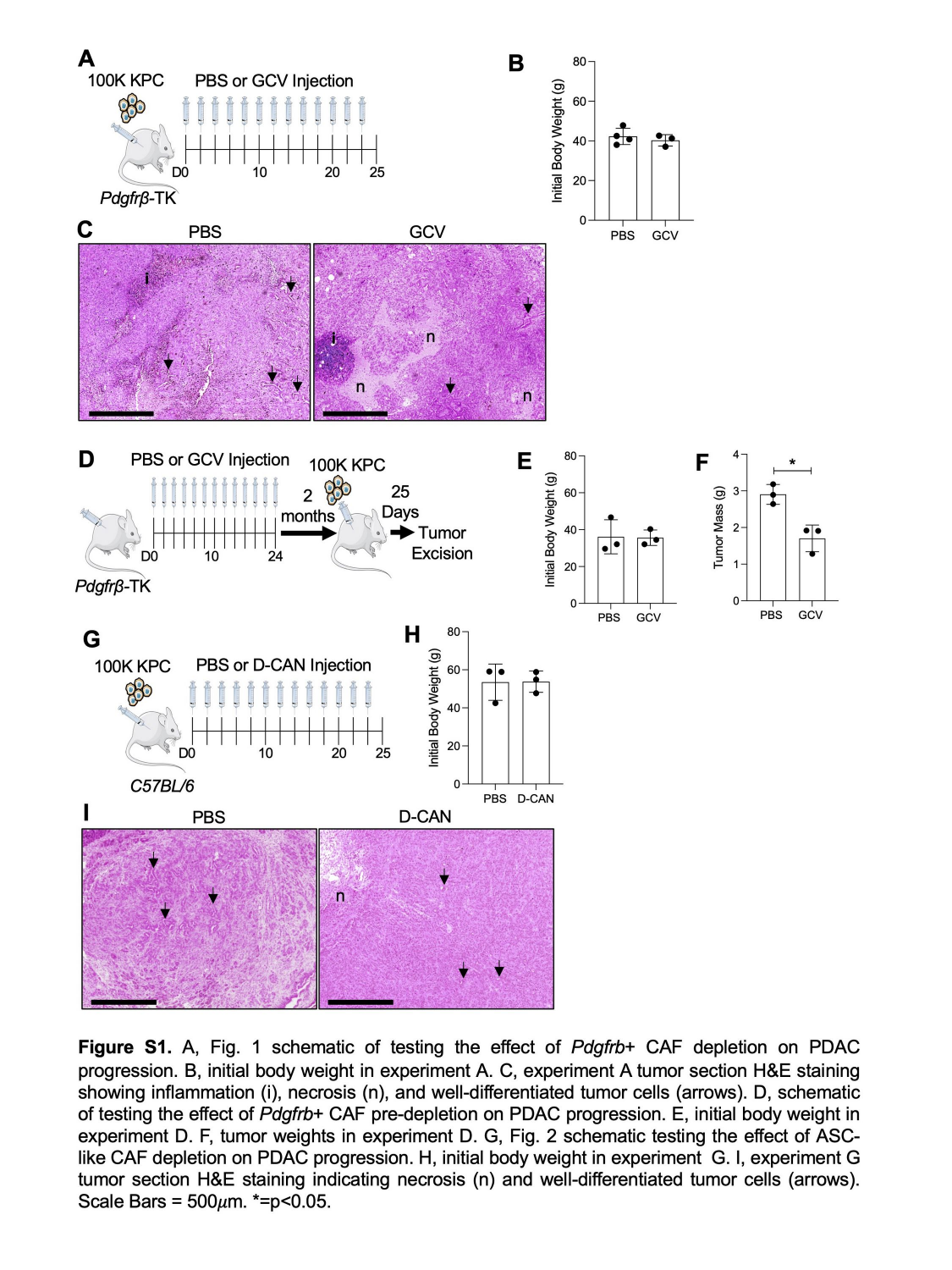

Supplement: Figure S1 — A, Fig. 1 schematic of testing the effect of Pdgfrb+ CAF depletion on PDAC progression. B, initial body weight in experiment A. C, experiment A tumor section H&E staining showing inflammation (i), necrosis (n), and well-differentiated tumor cells (arrows). D, schematic of testing the effect of Pdgfrb + CAF pre-depletion on PDAC progression. E, initial body weight in experiment D. F, tumor weights in experiment D. G, Fig. 2 schematic testing the effect of ASC-like CAF depletion on PDAC progression. H, initial body weight in experiment G. I, experiment G tumor section H&E staining indicating necrosis (n) and well-differentiated tumor cells (arrows). Scale Bars = 500μm. *=p<0.05. [file crc-24-0298_figure_s1_suppsf1.pptx]

## Slide 1
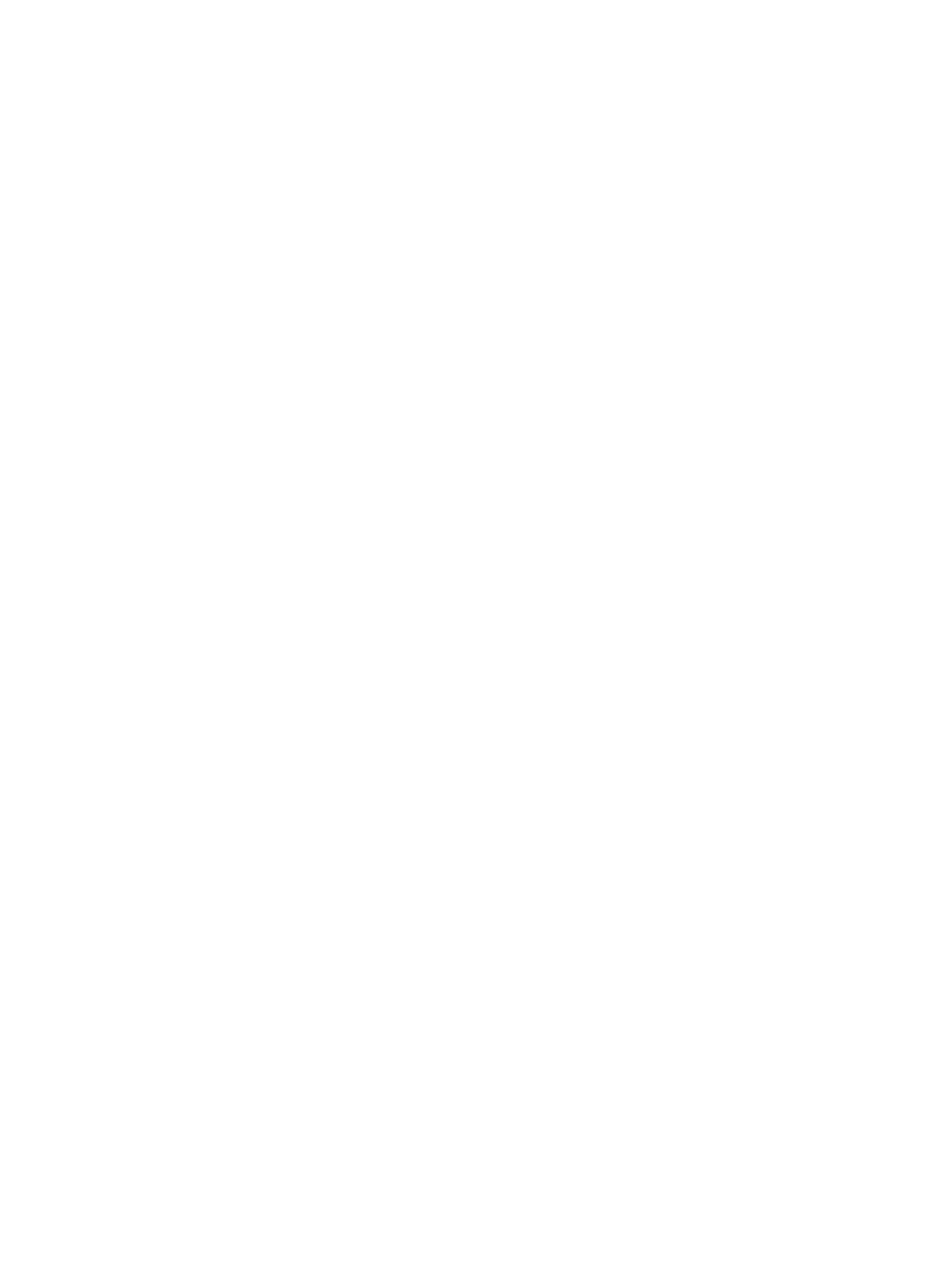

Supplement: Figure S2 — The effects of Pdgfrb+ CAF vs ASC-like CAF depletion on the TME. A, tumor section immunofluorescence for CD3 (Red) expression to measure T-cell infiltration and nuclei (blue) (left), CD3+ cell quantification (right). B, tumor section immunofluorescence for endomucin (green) to quantify endothelial cell density and nuclei (blue) (left), endomucin quantification (right). In A-B, for each tumor, a minimum of 15 fields (10X) were taken and quantified using Image J. Images were converted to 8-bit and a threshold was applied to only highlight endomucin expression and percent area was measured. An average was calculated for all images per tumor. Scale bar=100μm. *=p<0.05, ***=p<0.001.C, Volcano plots generated by IPA analysis of scRNAseq data in Fig. 3 identifying top genes induced (red) or suppressed (green) in KPC cells and CAFs from mice subjected to Pdgfrb+ CAF or ASC-like CAF depletion. D, Lists of the most upregulated (top) and downregulated (bottom) genes. [file crc-24-0298_figure_s2_suppsf2.pptx]

## Slide 1
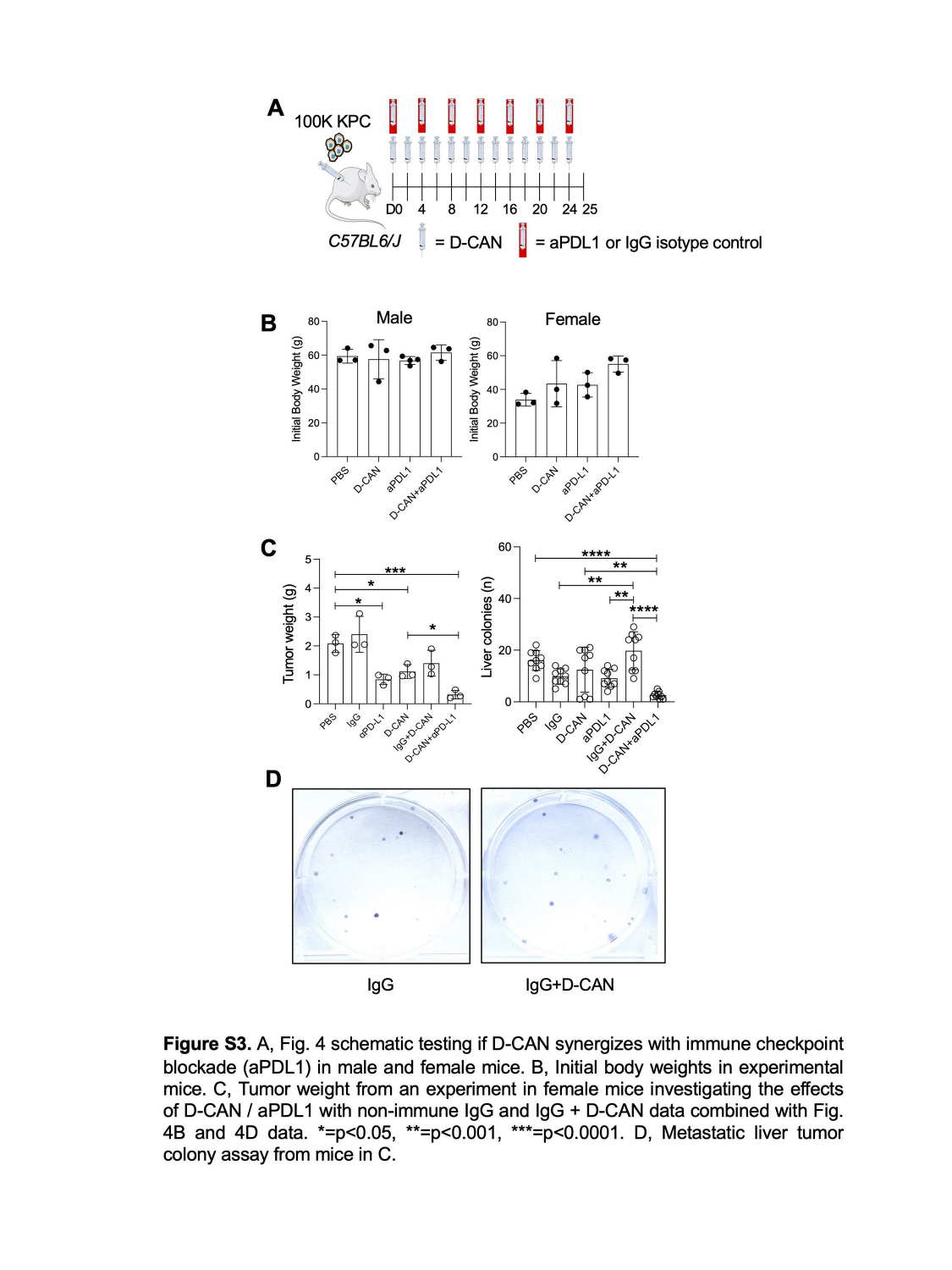

Supplement: Figure S3 — A, Fig. 4 schematic testing if D-CAN synergizes with immune checkpoint blockade (aPDL1) in male and female mice. B, Initial body weights in experimental mice. C, Tumor weight from an experiment in female mice investigating the effects of D-CAN / aPDL1 with non-immune IgG and IgG + D-CAN data combined with Fig. 4B and 4D data. *=p<0.05, **=p<0.001, ***=p<0.0001. D, Metastatic liver tumor colony assay from mice in C. [file crc-24-0298_figure_s3_suppsf3.pptx]
